# Investigations on Functional and Thermo-Mechanical Properties of Gluten Free Cereal and Pseudocereal Flours

**DOI:** 10.3390/foods11131857

**Published:** 2022-06-23

**Authors:** Iuliana Banu, Iuliana Aprodu

**Affiliations:** Faculty of Food Science and Engineering, Dunarea de Jos University of Galati, 111 Domneasca Str, 800008 Galati, Romania; iuliana.banu@ugal.ro

**Keywords:** gluten-free flours, solvent retention capacity, Mixolab, dough consistency

## Abstract

Seven commercial gluten-free (rice, oat, sorghum, foxtail millet, amaranth, quinoa, and buckwheat) flours were investigated in this study from the point of view of thermo-mechanical properties and solvent retention capacity (SRC). Each flour was used to prepare doughs with specific water absorption (WA) to get a consistency of 1.1 Nm (WA1) and doughs with WA2 levels higher than 85% to ensure a sufficient amount of water in the system for allowing the hydration of all components of the flours. Different correlations were established between proteins, ash, pentosans, damaged starch, and amylose contents on the one hand, and the capacity of the flour samples to retain different solvents such as sucrose, sodium carbonate and CaCl_2_ on the other hand. Although no significant correlation was found between the protein content of the flours and lactic acid-SRC, the mechanical weakening of the protein was significantly correlated with lactic acid-SRC for both tested WA levels. The doughs with WA1 had higher starch gelatinization and hot gel stability values compared to the corresponding dough systems with a higher water amount. Moreover, lower starch retrogradation and setback torques were obtained in the case of the dough prepared with higher amounts of water.

## 1. Introduction

The most suitable flour for obtaining gluten free baked products is rice flour. Rice is widely recognized as a hypoallergenic cereal with high nutritional value [1]. The proteins of both white and brown rice mainly consist of glutenin fraction, while the albumin, globulin, and gliadin fractions are in small quantities [1].

Generally, rice flour is blended with other grain flours to improve the functionality and nutritional properties of the final products [2].

In addition to rice, a large number of grains can be used to obtain gluten-free flour, such as the group of cereals named coarse cereals, which includes oat, sorghum, millet, and buckwheat, and the minor grain-like cereals, such as quinoa and amaranth. Beyond the gluten free property, every grain mentioned above is characterized by particular nutritional constituents.

Oats are recommended for their high content of β-glucans, which are components that have all the properties that are specific to water-soluble dietary fibers and are used as a healthy food ingredient [3,4]. Yue et al. [5] reported a high proportion of 7S globulin fraction in oat proteins.

Quinoa has high-quality proteins with a balanced amino acid content [6] and high levels of vitamin E that assures high stability of quinoa lipids during storage [7]. The quinoa proteins consist of 11S globulin fraction (37%), 2S albumin fraction (35%), while the prolamine fraction represents about 0.5–7% [8].

As well, amaranth is a good source of high-quality proteins [9], having a high content of methionine, cysteine, and lysine [10]. The distribution of protein fractions is closer to pulses than cereals and the digestibility of the proteins is high [10]. The proteins are mainly formed from albumins and globulins (50–60%) [9].

Buckwheat has proteins with a well-balanced amino acid composition and large amounts of flavonoids, rutin, and quercetin with good antioxidant activity [11]. The main protein fractions found in buckwheat are globulins (up 50%) and albumins (about 25%) [11].

Sorghum is rich in polyphenols and phytosterols [12], and the protein fractions mainly consist of kafirins [13].

Foxtail millet contains high levels of protein and fiber [14]. The main protein fractions from foxtail millet are prolamins (up to 60% of the total protein content) and glutelins, which are rich in essential amino acids [15]. Foxtail millet has been listed among protein sources with good potential for replacing animal proteins in different types of products. It has been reported that foxtail millet proteins display bioactive effects that are promising for the efficient management of different human chronic diseases [15].

In order to obtain gluten-free baked products with high overall quality, it is necessary to know the functionality of gluten-free flour constituents and the rheological properties of the dough. Starting from this, the objective of the study was to compare the physicochemical and functional properties of seven gluten-free flours as well as the thermo-mechanical properties of the dough systems.

## 2. Materials and Methods

### 2.1. Materials

Seven commercial gluten-free grain flours purchased from the Galati market (Romania) were used: rice flour (Solaris Plant SRL, Romania), oat flour (Sano Vita, Romania), sorghum flour (origin Hungary, distributed by Adams Vision SRL Tg Mures, Romania), foxtail millet flour (distributed by La Finestra sul Cielo Vilareggia, Italy), amaranth flour (Adams Vision SRL, Tg Mures, Romania), quinoa flour (Vitanescu Maricel, Romania), and buckwheat flour (distributed by SC Prifan Distribution SRL, Romania).

### 2.2. Proximate Analyses and Physical Properties

The chemical composition of the flour samples was determined as follows: moisture content by SR ISO 712:2005 [16], protein content using the semimicro-Kjeldahl method (Raypa Trade, R Espinar, SL, Barcelona, Spain), and the nitrogen-to-protein conversion factor of 5.95 for rice flours; 6.25 for oat, foxtail millet, buckwheat, and quinoa flours; 5.75 for sorghum flour; and 5.85 for amaranth flour. The fat content was determined through ether extraction using Soxhlet method (SER-148; VELP Scientifica, Usmate Velate (MB), Italy). The crude fiber content was determined by Fibretherm Analyser (C. Gerhardt GmbH & Co. KG, Königswinter, Germany). The total pentosans content was determined using the method described by Delcour et al. [17], and the ash content was determined by SR ISO 2171/2002 [16]. The starch content was estimated by subtracting 100 g of products from the average contents of the components that were determined experimentally. The Amylose/Amylopectin Assay Kit (Megazyne International Ireland Ltd. Wicklow, Ireland) was used to determine the amylose contents of the flours.

The damaged starch content was quantified using the AACC Method 76-31.01 [18] and the Starch Damage Assay Kit (Megazyme International Ireland Ltd. Wicklow, Ireland).

The fineness module was determined by sieving the flour samples through a 400, 315, 160, and 125 μm mesh [19].

### 2.3. Solvent Retention Capacity

The solvent retention capacity (SRC) profile was determined to be in agreement with the AACC Method 56-11.02 [18] when the percentage of solvents retained by the flour samples upon centrifugation for 15 min at 1000× *g*. The following solvents were tested: water (W-SRC), 5% sodium carbonate (SC-SRC), 50% sucrose (S-SRC), 5% lactic acid (LA-SRC) (AACC International, 2000), and 1 M CaCl2 (Ca-SRC) [3]. The SRC values were reported at moisture basis (14%).

### 2.4. Thermo-Mechanical Properties

The thermo-mechanical properties of the flours were determined through the Chopin+ protocol using the Mixolab device (Chopin Technology, Villeneuve La Garenne, France). To better understand the evolution of dough during thermal and mixing constraints, two water absorption (WA) values were considered when running the Chopin+ protocol on each of the tested flours. WA1 was needed to obtain dough with a maximum torque C1 of 1.1 Nm, except for sorghum and buckwheat, for which it was not possible to reach the targeted dough consistency and WA2 of 85%. Oat flour was the only sample for which the C1 of 1.1 Nm was achieved for WA1 of 85%. The following torques were registered in the case of the Mixolab tests performed at both WA levels: maximum C1 torque at initial mixing, consistency of the dough after 8 min of mixing at constant temperature of 30 °C (CS), C2 showing dough changes at heating caused by protein weakening, C3 associated to starch gelatinization, C4 provided information on the stability of the hot gel, and C5 registered the cooling phase when starch retrogradation occurred [20]. Further thermo-mechanical indicators were calculated as follows: mechanical weakening of the proteins MWP = (C1 − CS)/C1 × 100, strength of the protein network while heating the dough (C1-C2), intensity of starch gelatinization (C3-C2), breakdown torque (C3-C4), and setback torque (C5-C4) [21].

### 2.5. Statistical Analysis

Triplicate measurements were performed, and the results are presented as the average ± standard deviation values. The significant differences among samples were assessed using the Minitab 19 software (Minitab Inc., State College, PA, USA) through the one-way ANOVA with a 95% confidence interval, after assessing the normality and variance equality conditions. The Tukey method was selected for the post-hoc analysis when *p* values lower than 0.05 were indicated by ANOVA analysis. The Pearson’s correlation was calculated to identify the potential relationships between the SRC of the flour and dough characteristics.

## 3. Results and Discussion

### 3.1. Proximate Compositions and Physical Properties of Gluten-Free Flours

The proximate composition of the gluten-free flours is shown in Table 1. The protein content varied from 6.21% in rice flour to 13.98% in quinoa flour. Overall, higher protein contents were found in pseudocereals, i.e., quinoa, amaranth, and buckwheat, compared to cereals, i.e., rice, millet, sorghum, and oat.

The highest contents of starch were registered in rice, sorghum, and oat flour, while millet flour had the lowest starch content. The fiber content ranged from 16.33% in millet flour to 3.72% in sorghum flour. However, oat flour had the highest pentosans content of 5.13%, while rice and amaranth flour had the lowest values of 1.41–1.48%. Amaranth, millet, and quinoa flour had the highest fat (6–5.30%) and ash (2.94–2.41%) content, while rice flour had the lowest fat (2.14%) and ash (1.51%) content.

The damaged starch content ranged between 1.58%, in the case of buckwheat flour, and 5.29%, in the case of oat flour. The starch damage is the result of ripping, rubbing, shearing, and cutting forces acting on the grains during the milling process [3], but the extent of the damage also depends on the endosperm structure [22,23]. Therefore, even if oat and quinoa flour had close values for the fineness module—2.26% and 2.25%, respectively—they had significantly different damaged starch contents of 5.29% and 2.71%, respectively (Table 1).

### 3.2. Solvent Retention Capacity of Gluten-Free Flours

SRC was initially developed to define the functional profile of wheat flour to allow for the prediction of the baking performance of flour [18]. In the last few years, SRC has been also used for characterizing different gluten-free flours [24,25].

The SRC values of the gluten-free flours investigated in the present study are reported in Table 2. The W-SRC varied from 87.00% to 126.34%, with the lowest value being registered for quinoa flour, while the highest was for amaranth flour. However, the W-SRC values of all investigated flours are much higher than those indicated by the AACC method 56-11.02 [18] for the wheat flour recommended for cookies (W-SRC below 51%) or sponge and dough bread (W-SRC below 57%).

SC-SRC ranged from 94.00% to 122.62%. According to Kweon et al. [26], SC-SRC is related to the content of damaged starch, which is easily soluble in Na_2_CO_3_ solution with a pH above the pKa of the starch hydroxyl groups. Indeed, oat flour, having the highest level of damaged starch (5.29%), exhibited the highest SC-SRC value, while buckwheat flour, with the lowest value of damaged starch (1.58%), presented the lowest SC-SRC value (Table 2). In agreement with the findings of Kweon et al. [26], the content of damaged starch for the gluten-free flours was significantly correlated (R^2^ of 0.938 and *p* < 0.01) with SC-SRC, confirming the high swelling ability of the damaged starch when placed in contact with Na_2_CO_3_ solution. In addition, a significant correlation (R^2^ of 0.758 and *p* < 0.05) was found between the ash content (Table 1) and SC-SRC (Table 2) of the investigated gluten-free flours.

Oat flour had the highest S-SRC value of 145.41% among all investigated gluten-free flours. In addition, as indicated in Table 2, oat flour had the highest pentosans content. In fact, a significant correlation (R^2^ of 0.964 of *p* < 0.01) was found between pentosans content and S-SRC.

LA-SRC provides information on gluten functionality, being particularly related to glutenin characteristics. Anyway, the presence of high amounts of bran particles, which have good swelling ability in lactic acid solution, might interfere with the accurate interpretation of the LA-SRC of whole flours [27]. If in the case of wheat flour the LA-SRC varied generally between 100–115% [27], in case of the gluten-free flour investigated in our study, the LA-SRC varied from 114.81% to 134.72%. The correlation (R^2^ of 0.308 and *p* < 0.05) between the protein content and LA-SRC was not significant.

A significant correlation (R^2^ of 0.758 and *p* < 0.05) was found between the ash content of gluten-free flours and Ca-SRC. Additional correlations were registered between the protein contents and Ca-SRC (R^2^ of 0.852 and *p* < 0.05), and between the amylose content and Ca-SRC (R^2^ of 0.920 and *p* < 0.01). On the other hand, in the case of oat flour, different authors reported significant correlations between Ca-SRC and β-glucan, with an R^2^ of 0.615 (*p* < 0.01) [4] and R^2^ of 0.82 (*p* < 0.01), respectively [3]. Additionally, Zhang et al. [4] reported a significant correlation between Ca-SRC and the molecular weight of β-glucan of oat flour (R^2^ of 0.366 and *p* < 0.05). As indicated by Guo et al. [28] and Yamazaki et al. [29], the water retention capacity of β-glucans is favored upon binding metal ions like Ca^2+^ in a solution with pH that is regulated from neutral to acidic.

### 3.3. The Thermo-Mechanical Properties

**Figure 1:**
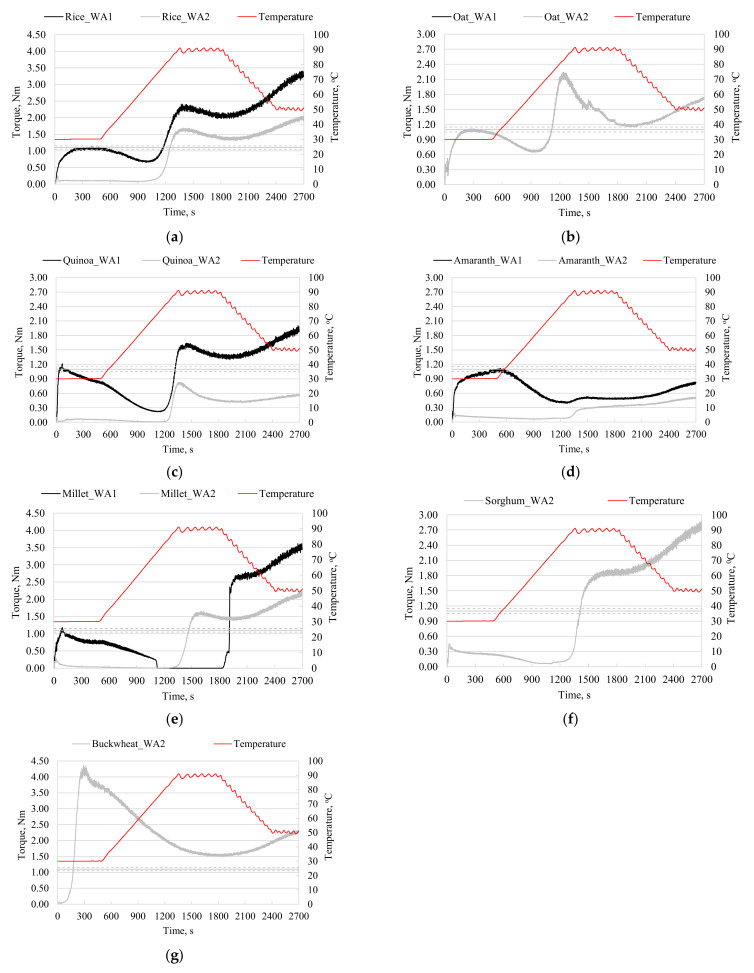
Mixolab curves of the dough samples prepared with rice (**a**), oat (**b**), quinoa (**c**), amaranth (**d**), millet (**e**), sorghum (**f**), and buckwheat (**g**) flour at different water absorption (WA) levels: WA1 necessary to obtain a maximum torque of 1.1 ± 0.05 Nm and WA2 of 85%. Note: For the oat flour, WA1 and WA2 had the same value of 85%.

The Mixolab device was designed for the investigation of wheat dough properties during the dual constraints of kneading and temperature. If in the case of the wheat flour bread the dough consistency of 1.1 ± 0.05 Nm is a benchmark, in the case of gluten-free flours, this dough consistency is no longer a necessary target, given the different technology for preparing bread in the absence of gluten in the system. Cappa et al. [30] appreciated that, in the case of gluten-free formulas, a lower dough consistency is preferred. For this reason, two experimental set-ups were considered in the present study, which look for the investigation of gluten-free-flour-based dough rheology at specific water absorption levels that are needed to obtain a dough consistency of 1.1 ± 0.05 Nm (WA1) in the case of each investigated flour (Table 3), and at the same water absorption of 85% (WA2), which is chosen in such a manner as to assure a dough consistency value below 1.1 Nm (Table 4). In Figure 1, the Mixolab curves are depicted, showing substantial differences between the thermo-mechanical profiles of the investigated gluten-free flours. The particularities of the thermo-mechanical behavior of the gluten-free doughs are derived from the differences in the chemical composition and hydration ability of the flour components during kneading, and the further dough behavior during heating and cooling.

As can be seen from Table 3, the water absorption needed to obtain dough with 1.1 ± 0.05 Nm varied in large limits. Moreover, in the case of sorghum and buckwheat flour, it was not possible to reach 1.1 ± 0.05 Nm during kneading at constant temperature (30 °C). Torbica et al. [31] highlighted the importance of the amount of water used for preparing gluten-free dough. They reported the higher water requirements for preparing the oat flour-based dough. After analyzing the results presented in Table 3, one can see that, among the seven investigated gluten-free flours, the highest water absorption was registered for oat flour. Our observations regarding the behavior of the sorghum flour comply with the findings of Torbica et al. [31], who failed to obtain dough with a consistency of 1.1 Nm. The sorghum-flour-based dough was very firm and remained attached to the arms of the mixer in such a manner that the device could not record the consistency values.

In order to compare the strength of the dough during kneading, MWP values were calculated (Table 4). The low MTW values are associated with a high value of the torque after 8 min of kneading, which means there is a higher stability for dough during kneading. Doughs prepared with rice, amaranth, and oat flour exhibited higher resistance during kneading compared to the millet and quinoa doughs. These results can be explained by the presence of different types of protein fractions that are present in the gluten-free flours and their particular behavior during kneading. The main proteins found in the rice flour are glutenins [1], while for the oat flour, globulins are the most abundant fraction [5]. An important additional factor that influences the C1 values is the pentosans content (5.13% in oat flour and 1.48% in rice flour).

As the temperature rises, the dough consistency decreases until it reaches the C2 value. The rice and oat doughs had (C1-C2) values close to those of wheat dough, while for the amaranth, quinoa, and millet doughs, the (C1-C2) values were much higher. The same C2 value of 0.67 Nm was registered for the oat flour at both tested WA levels, whereas for rice flour, C2 decreased from 0.68 to 0.09 Nm when WA increased from 66% to 85% (Table 3 and Table 4). Even if quinoa, amaranth, and millet flour are able to form doughs with C1 of 1.1 ± 0.05 Nm at similar WA levels of 61–62.1% (Table 3), the C2 values varied significantly (*p* < 0.05). Amaranth flour presented a C2 value that was about two times higher than quinoa flour, while the lowest C2 value was registered for millet flour, suggesting the weakness of the protein network during heating. In addition to the higher protein contents in quinoa and amaranth flour compared to millet flour, the protein fractions prevailing in quinoa and amaranth flour, consisting of albumins and globulins, have good solubility in water and dilute salt solutions [32]—unlike prolamins and glutelins, which are mainly found in millet flour and have poor solubility [33].

When comparing the C2 of rice and amaranth flour-based doughs, Hadnadev et al. [20] noted that the lower weakening of rice flour proteins is due to mechanical and thermal constraints, whereas the high weakening observed for amaranth flour, which had a higher protein content, was assigned to the lower protein quality.

Doughs prepared with WA 85% can be distributed in three groups based on the behavior during kneading at a constant temperature (30 °C) and while heating up to 50–55 °C: (i) oat dough characterized by a Mixolab curve (Figure 1b) was similar to those specific to wheat flour; (ii) rice (Figure 1a), quinoa (Figure 1c), amaranth (Figure 1d), millet (Figure 1e), and sorghum (Figure 1f) dough with C1 below 1.1 ± 0.05 Nm and C2 values falling in a very narrow range of 0.01–0.09 Nm; and (iii) buckwheat dough with a Mixolab curve having C1 over 4.50 Nm and lacking C2, with the dough consistency being in a continuous decrease during heating from 30 °C to 50–55 °C (Table 4, Figure 1g). For gluten-free flour from group (ii), the water amount from the dough system appears to be too high with respect to the requirements for the chemical components of the flour, while in the case of buckwheat flour, the amount of water used for preparing the dough appeared to be too low.

MWP was significantly correlated with the LA-SRC for both tested WA levels: WA1 needed to obtain C1 of 1.1 ± 0.05 Nm (R^2^ of 0.891, *p* < 0.05) and WA2 of 85% (R^2^ of 0.719 and *p* < 0.05).

Additional correlations were established between the various solvent retention capacities of the gluten-free flours and the thermo-mechanical properties of the corresponding doughs, which depended on the WA level. For instance, for WA1, the C2 (R^2^ of 0.941 and *p* < 0.01), (C2-C1) (R^2^ of 0.943 and *p* < 0.01), and MWP (R^2^ of 0.776 and *p* < 0.05) were significantly correlated with SC-SRC. Moreover, for WA1, the Ca-SRC was significantly correlated with (C1-C2) (R^2^ of 0.968 and *p* < 0.01), while for WA2, Ca-SRC was significantly correlated with MWP (R^2^ of 0.629 and *p* < 0.05). According to Codină et al. [34], calcium ions decrease the softening degree of the dough during kneading. For wheat flour, Ca^2+^ was reported to improve protein solubility and to favor the overall hydration capacity [35]. When factoring in the role played by wheat flour starch, the destabilization effect, which is associated in particular with the damaged starch, should be considered. Given the existence of high spaces between the amylopectin chains in the damaged starch of wheat flour, the binding of Ca^2+^ is facilitated, thereby favoring the increase of the water absorption values of the doughs.

Finally, (C1-C2) was correlated with LA-SRC (R^2^ of 0.684 and *p* < 0.05) when the WA2 of 85% was used, suggesting that the ability of the gluten-free flours to retain the lactic acid solution might provide information on the weakening behavior of the proteins.

As the temperature rises from 50–55 °C to 90 °C, the protein’s contribution to the dough consistency decreases and the starch properties become more important. Higher starch gelatinization (C3) and hot gel stability (C4) values were observed for the doughs prepared using WA1, which needed to obtain C1 of 1.1 ± 0.05 Nm, compared to WA2 of 85% (Table 3 and Table 4). The use of a higher WA level of 85% appeared to benefit the dough by improving the structure of the dough prepared with millet, oat, and rice, respectively. Sorghum, buckwheat, and millet formed stronger starch networks than rice and oat when the WA2 of 85% was used.

In the case of quinoa and amaranth flour, the dough with WA2 had a very low maximum consistency C3 and starch retrogradation (C5-C4) (Table 4) compared to the corresponding dough samples prepared with WA1. The Mixolab curve indicated an atypical behavior of the amaranth flour-based dough at 95 °C and while cooling at 50 °C compared to other investigated flour samples. Similar results were reported by Inglett et al. [36], who obtained low values for the maximum viscosity, and the viscosity remained constant even after cooling to 50 °C.

Starch behavior is influenced by the particularities of the starch structure; more specifically, it is influenced by the length of the amylose and amylopectin chains and the ratio between the two macromolecules [37]. Quinoa and amaranth have amylose contents of 10.92% and 17.96%, respectively. Although millet has an intermediate amylose content of 12.77%, a different starch retrogradation behavior was noticed, which was likely the result of differences in the botanical source of the starch. It should also be noted that quinoa, amaranth, and millet had a lower content of starch and higher content of lipids compared to the other investigated flours. These observations comply with previous findings that indicate that lipids can complex amylose, thereby causing a reduction in the peak viscosity [21,38]. On the other hand, rice flour, which has the highest amount of carbohydrates among all investigated flours (Table 1), had maximum peak torque (C3) and gelatinization intensity (C3-C2) values that prevail over those of other flours when the doughs were prepared using WA1, which was needed to obtain C1 of 1.1 ± 0.05 Nm.

The dough systems prepared with a higher amount of water (WA2) presented a lower starch retrogradation (C5) compared to the corresponding doughs with WA1. A better bread-making performance of the flours with a low C5 value was suggested by Ekpa et al. [39], who also related these parameters with a slow staling process. The increase of the water level used to prepare the dough, from WA1 to WA2, also resulted in a decrease of the setback torques (C5-C4) (Table 3 and Table 4). In agreement with Ekpa et al. [39], this decrease might result in the improvement of the shelf life of bread.

## 4. Conclusions

Significant correlations were found between the solvent retention capacity of gluten-free flours and the thermo-mechanical properties of the doughs prepared at two different hydration levels. A significant correlation was found at lower hydration levels between the mechanical weakening of the proteins and the LA-SRC. At a high water absorption of 85%, the strength of the protein network while heating the dough was correlated with LA-SRC, but no correlation could be established at a lower water absorption level when the dough had C1 of 1.1 ± 0.05 Nm. The starch behavior at high temperatures highly depended on the amount of water used to prepare the dough systems. The gluten-free dough systems with a higher water absorption presented higher values of starch gelatinization and hot gel stability. The high amount of water in the dough system also resulted in the decrease of starch retrogradation and setback torques, a situation that can be associated with the increase of bread shelf life.

## Figures and Tables

**Table 1:** Proximate compositions and fineness modules of the gluten free flours.

Component	Flours						
	Rice	Oat	Sorghum	Millet	Buckwheat	Quinoa	Amaranth
Moisture, %	11.28 ± 0.03 ^b^	10.9 ± 0.05 ^c^	8.16 ± 0.01 ^e^	10.91 ± 0.03 ^c^	11.28 ± 0.02 ^b^	10.25 ± 0.02 ^d^	11.69 ± 0.03 ^a^
Ash, %	1.51 ± 0.01 ^f^	1.42 ± 0.01 ^g^	1.61 ± 0.01 ^e^	2.68 ± 0.02 ^b^	1.88 ± 0.01 ^d^	2.41 ± 0.01 ^c^	2.94 ± 0.01 ^a^
Protein, %	6.21 ± 0.04 ^f^	10.91 ± 0.07 ^d^	9.81 ± 0.02 ^e^	9.85 ± 0.02 ^e^	11.60 ± 0.03 ^c^	13.98 ± 0.02 ^a^	13.59 ± 0.02 ^b^
Fat, %	2.14 ± 0.03 ^g^	3.88 ± 0.03 ^d^	3.18 ± 0.02 ^e^	5.72 ± 0.02 ^b^	2.67 ± 0.03 ^f^	5.30 ± 0.02 ^c^	6.00 ± 0.02 ^a^
Crude fiber, %	6.60 ± 0.02 ^d^	7.19 ± 0.02 ^c^	3.72 ± 0.05 ^f^	16.33 ± 0.06 ^a^	10.22 ± 0.03 ^b^	5.31 ± 0.02 ^e^	6.70 ± 0.03 ^d^
Pentosans, %	1.48 ± 0.02 ^f^	5.13 ± 0.03 ^a^	3.71 ± 0.02 ^c^	4.45 ± 0.02 ^b^	3.56 ± 0.01 ^d^	2.22 ± 0.03 ^e^	1.41 ± 0.01 ^g^
Starch, %	83.54 ± 0.05 ^a^	76.60 ± 0.11 ^c^	81.68 ± 0.08 ^b^	65.42 ± 0.06 ^g^	73.63 ± 0.06 ^d^	73.00 ± 0.06 ^e^	70.77 ± 0.02 ^f^
Damaged starch, %	4.40 ± 0.05 ^b^	5.29 ± 0.04 ^a^	3.79 ± 0.03 ^d^	3.20 ± 0.02 ^e^	1.58 ± 0.02 ^g^	2.71 ± 0.02 ^f^	3.96 ± 0.01 ^c^
Amylose, %	31.32 ± 0.30 ^a^	27.64 ± 0.26 ^b^	21.79 ± 0.20 ^d^	12.77 ± 0.25 ^f^	23.52 ± 0.36 ^c^	10.92 ± 0.26 ^g^	17.90 ± 0.35 ^e^
Fineness module	2.85 ± 0.05 ^a^	2.26 ± 0.05 ^c^	1.55 ± 0.05 ^e^	1.87 ± 0.03 ^d^	2.36 ± 0.05 ^b,c^	2.25 ± 0.05 ^c^	2.46 ± 0.05 ^b^

Means from the same row not sharing a superscript letter are significantly different (*p* < 0.05).

**Table 2:** Solvent retention capacity of gluten-free flours.

SRC	Flours						
	Rice	Oat	Sorghum	Millet	Buckwheat	Quinoa	Amaranth
W-SRC, %	101.96 ± 0.48 ^e^	115.23 ± 0.50 ^c^	115.61 ± 0.44 ^c^	107.28 ± 0.54 ^d^	121.06 ± 0.41 ^b^	87.00± 0.36 ^f^	126.34 ± 0.57 ^a^
Ca-SRC, %	142.82 ± 0.20 ^a^	133.25 ± 0.25 ^b^	131.06 ± 0.48 ^c^	118.74 ± 0.25 ^e^	122.52 ± 0.28 ^d^	104.16 ± 0.30 ^g^	117.75 ± 0.31 ^f^
SC-SRC, %	116.52 ± 0.50 ^b^	122.62 ± 0.47 ^a^	106.28 ± 0.20 ^d^	96.52 ± 0.30 ^e^	94.00 ± 0.30 ^f^	96.56 ± 0.23 ^e^	109.40 ± 0.36 ^c^
LA-SRC, %	132.29 ± 0.26 ^b^	124.99 ± 0.34 ^d^	126.40 ± 0.36 ^c^	119.32 ± 0.20 ^e^	134.72 ± 0.37 ^a^	114.81 ± 0.20 ^f^	131.83 ± 0.32 ^b^
S-SRC, %	125.00 ± 0.36 ^f^	145.41 ± 0.37 ^a^	136.40 ± 0.36 ^c^	142.06 ± 0.31 ^b^	135.11 ± 0.35 ^d^	130.15 ± 0.22 ^e^	117.08 ± 0.19 ^g^

W-SRC—water retention capacity; Ca-SRC—CaCl_2_ solvent retention capacity; Na-SRC—NaCl solvent retention capacity; SC-SRC—sodium carbonate solvent retention capacity; LA-SRC—lactic acid solvent retention capacity; Su-SRC—sucrose solvent retention capacity. Means from the same row not sharing a superscript letter are significantly different (*p* < 0.05).

**Table 3:** The thermo-mechanical properties of gluten-free flours at specific water absorption (WA) levels required to obtain doughs with the maximum consistency C1 of 1.1 ± 0.05 Nm.

Parameters	Flours				
	Rice	Oat	Quinoa	Amaranth	Millet
WA, %	66.0	85.0	62.1	61.0	61.9
C1, Nm	1.08 ± 0.03 ^b^	1.09 ± 0.01 ^b^	1.15 ± 0.01 ^a^	1.08 ± 0.01 ^b^	1.04 ± 0.01 ^c^
CS, Nm	1.06 ± 0.03 ^a^	1.03 ± 0.01 ^a^	0.83 ± 0.01 ^b^	1.06 ± 0.01 ^a^	0.76 ± 0.01 ^c^
C2, Nm	0.68 ± 0.01 ^a^	0.67 ± 0.01 ^a^	0.22 ± 0.01 ^c^	0.41 ± 0.01 ^b^	0.15 ± 0.01 ^d^
C3, Nm	2.31 ± 0.01 ^a^	2.18 ± 0.01 ^b^	1.60 ± 0.02 ^c^	0.51 ± 0.01 ^d^	nd
C4, Nm	2.04 ± 0.02 ^b^	1.17 ± 0.02 ^d^	1.35 ± 0.01 ^c^	0.49 ± 0.01 ^e^	2.67 ± 0.01 ^a^
C5, Nm	3.33 ± 0.04 ^b^	1.73 ± 0.02 ^d^	1.92 ± 0.01 ^c^	0.81 ± 0.01 ^e^	3.54 ± 0.01 ^a^
MWP, %	1.85 ± 0.05 ^c^	5.50 ± 0.05 ^b^	27.83 ± 0.24 ^a^	1.85 ± 0.01 ^c^	26.93 ± 1.43 ^a^
C1-C2, Nm	0.40 ± 0.03 ^c^	0.42 ± 0.02 ^c^	0.93 ± 0.01 ^a^	0.67 ± 0.02 ^b^	0.89 ± 0.02 ^a^
C3-C2, Nm	1.63 ± 0.01 ^a^	1.51 ± 0.01 ^b^	1.38 ± 0.03 ^c^	0.10 ± 0.00 ^d^	nd
C3-C4, Nm	0.27 ± 0.03 ^b^	1.01 ± 0.02 ^a^	0.25 ± 0.03 ^b^	0.02 ± 0.01 ^c^	nd
C5-C4, Nm	1.29 ± 0.04 ^a^	0.56 ± 0.00 ^c^	0.57 ± 0.02 ^c^	0.32 ± 0.02 ^d^	0.87 ± 0.01 ^b^

Means from the same row not sharing a superscript letter are significantly different (*p* < 0.05); nd—not detected; MWP—mechanical weakening of the proteins.

**Table 4:** The thermo-mechanical properties of gluten-free flours at a water absorption of 85% used for obtaining doughs.

Parameters	Flours						
	Rice	Oat	Quinoa	Amaranth	Millet	Sorghum	Buckwheat
C1, Nm	0.11 ± 0.01 ^f^	1.09 ± 0.01 ^b^	0.81 ± 0.01 ^c^	0.14 ± 0.01 ^f^	0.21 ± 0.02 ^e^	0.41 ± 0.01 ^d^	4.19 ± 0.01 ^a^
CS, Nm	0.10 ± 0.01 ^d^	1.03 ± 0.01 ^b^	0.06 ± 0.01 ^e^	0.09 ± 0.01 ^d^	0.04 ± 0.01 ^e^	0.24 ± 0.01 ^c^	3.68 ± 0.01 ^a^
C2, Nm	0.09 ± 0.01 ^b^	0.67 ± 0.01 ^a^	0.01 ± 0.01 ^d^	0.08 ± 0.01 ^b,c^	0.01 ± 0.01 ^d^	0.06 ± 0.01 ^c^	nd
C3, Nm	1.64 ± 0.02 ^c^	2.18 ± 0.01 ^a^	0.80 ± 0.02 ^d^	0.28 ± 0.01 ^e^	1.60 ± 0.02 ^c^	1.85 ± 0.01 ^b^	nd
C4, Nm	1.36 ± 0.01 ^d^	1.17 ± 0.02 ^e^	0.43 ± 0.02 ^f^	0.31 ± 0.01 ^g^	1.42 ± 0.01 ^c^	1.87 ± 0.02 ^a^	1.53 ± 0.01 ^b^
C5, Nm	1.98 ± 0.01 ^d^	1.73 ± 0.01 ^e^	0.42 ± 0.01 ^g^	0.51 ± 0.02 ^f^	2.15 ± 0.00 ^c^	2.77 ± 0.02 ^a^	2.28 ± 0.02 ^b^
MWP, %	9.14 ± 0.83 ^e,f^	5.50 ± 0.05 ^f^	92.60 ± 1.14 ^a^	35.84 ± 2.57 ^d^	81.14 ± 2.98 ^b^	41.48 ± 1.01 ^c^	12.17 ± 0.45 ^e^
C1-C2, Nm	0.02 ± 0.01 ^e^	0.42 ± 0.02 ^b^	0.80 ± 0.01 ^a^	0.06 ± 0.01 ^e^	0.20 ± 0.03 ^d^	0.35 ± 0.02 ^c^	nd
C3-C2, Nm	1.55 ± 0.02 ^b,c^	1.51 ± 0.01 ^c^	0.79 ± 0.03 ^d^	0.20 ± 0.01 ^e^	1.59 ± 0.03 ^b^	1.79 ± 0.00 ^a^	nd
C3-C4, Nm	0.28 ± 0.01 ^c^	1.01 ± 0.02 ^a^	0.37 ± 0.03 ^b^	−0.03 ± 0.02 ^e^	0.18 ± 0.01 ^d^	−0.02 ± 0.01 ^e^	nd
C5-C4, Nm	0.62 ± 0.00 ^c^	0.56 ± 0.01 ^d^	−0.01 ± 0.01 ^f^	0.20 ± 0.03 ^e^	0.73 ± 0.01 ^b^	0.90 ± 0.00 ^a^	0.75 ± 0.01 ^b^

Means from the same row not sharing a superscript letter are significantly different (*p* < 0.05); nd—not detected; MWP—mechanical weakening of the proteins.

## Data Availability

Data is contained within the article.

## References

[1] Cao X., Wen H., Li C., Gu Z. (2009). Differences in functional properties and biochemical characteristics of congenetic rice proteins. J. Cereal Sci..

[2] Badiu E., Aprodu I., Banu I. (2014). Trends in the development of gluten-free bakery products. Nnals Univ. Dunarea De Jos Galati Fasc. VI-Food Technol.

[3] Niu Q., Pu Y., Li X., Ma Z., Hu X. (2017). Solvent Retention Capacities of Oat Flour. Int. J. Mol. Sci..

[4] Zhang K., Li X., Ma Z., Hu Z. (2019). Solvent retention capacity of oat flour: Relationship with oat β-glucan content and molecular weight. Food Hydrocoll..

[5] Yue J., Gu Z., Zhu Z., Yi J., Ohm J.B., Chen B., Rao J. (2021). Impact of defatting treatment and oat varieties on structural, functional properties, and aromatic profile of oat protein. Food Hydrocoll..

[6] Agrawal R.S. (2018). Quinoa—Supergrain of the future: A Review. Pharma Innov..

[7] Ng S.C., Anderson A., Coker J., Ondrus M. (2007). Characterization of lipid oxidation products in quinoa (*Chenopodium quinoa*). Food Chem..

[8] Dakhili S., Abdolalizadeh L., Hosseini S.M., Shojaee-Aliabadi S., Mirmoghtadaie L. (2019). Quinoa protein: Composition, structure and functional properties. Food Chem..

[9] Joshi D.C., Sood S., Hosahatti R., Kant L., Pattanayak A., Kumar A., Yadav D., Stetter M.G. (2018). From zero to hero: The past, present and future of grain amaranth breeding. Theor. Appl. Genet..

[10] Narwade S., Pinto S. (2018). Amaranth—A Functional Food. Concepts Dairy Vet. Sci..

[11] Torbica A., Hadnađev M., Hadnađev T.D. (2012). Rice and buckwheat flour characterisation and its relation to cookie quality. Food Res. Int..

[12] Fu J., Zhang Y., Hu Y., Zhao G., Tang Y., Zou L. (2020). Concise review: Coarse cereals exert multiple beneficial effects on human health. Food Chem..

[13] Hamaker B.R., Bugusu B.A. Overview: Sorghum protein and food quality. Department of Food Science. Purdue University and the INTSORMIL CRSP West Lafayette, Indiana, USA (20 May 2003). Proceedings of the Afripro-Workshop on the Proteins of Sorghum and Millets: Enhancing Nutritional and Functional Properties for Africa.

[14] Sharma N., Niranjan K. (2018). Foxtail millet: Properties, processing, health benefits, and uses. Food Rev. Int..

[15] Sachdev N., Goomer S., Singh L.R. (2021). Foxtail millet: A potential crop to meet future demand scenario for alternative sustainable protein. J. Sci. Food Agr..

[16] (2008). SR ISO 712:2005; SR ISO 2171/2002.

[17] Delcour J.A., Vanhamel S., De Geest C. (1989). Physico-Chemical and Functional Properties of Rye Nonstarch Polysaccharides. I. Colorimetric Analysis of Pentosans and Their Relative Monosaccharide Compositions in Fractionated (Milled) Rye Products. Cereal Chem..

[18] AACC International (2000). Approved Methods of Analysis.

[19] Godon B., Wilhm C. (1994). Primary Cereal Processing A Comprehensive Sourcebook.

[20] Dubat A., Boinot N. (2012). Mixolab Applications Handbook. Rheological and Enzymes Analyses.

[21] Hadnadev T.D., Torbica A., Hadnadev M. (2011). Rheological properties of wheat flour substitutes/alternative crops assessed by Mixolab. Procedia Food Sci..

[22] Barrera G., Perez G., Ribotta P., Leon A. (2007). Influence of damaged starch on cookie and bread-making quality. Eur. Food Res. Technol..

[23] Topin V., Radjai F., Dellene J.Y., Sadoudi A., Mabille F. (2008). Wheat endosperm as a cohesive granular material. J. Cereal Sci..

[24] Collar C., Angioloni A. (2014). Pseudocereals and teff in complex breadmaking matrices: Impact on lipid dynamics. J. Cereal Sci..

[25] Mariotti M., Lucisano M., Pagani M.A., Ng P.K.W. (2016). Effects of dispersing media and heating rates on pasting profiles of wheat and gluten-free samples in relation to their solvent retention capacities and mixing properties. LWT Food Sci. Technol..

[26] Kweon M., Slade L., Levine H. (2011). Solvent retention capacity (SRC) testing of wheat flour: Principles and value in predicting flour functionality in different wheat-based food processes and in wheat breeding—A review. Cereal Chem..

[27] Wang N., Hou G.G., Kweon M., Lee B. (2016). Effects of particle size on the properties of whole-grain soft wheat flour and its cracker baking performance. J. Cereal Sci..

[28] Guo X., Hu G., Liu S. (2001). Anti-nutritive role of oat β-glucan and application of oat β-glucanase in feed. Jiangxi Feed.

[29] Yamazaki E., Murakami K., Kurita O. (2005). Easy preparation of dietary fiber with the high water-holding capacity from food sources. Plant Foods Hum. Nutr..

[30] Cappa C., Lucisano M., Mariotti M. (2013). Influence of Psyllium, sugar beet fibre and water on gluten-free dough properties and bread quality. Carbohyd. Polym..

[31] Torbica A., Belović M., Tomić J. (2019). Novel breads of non-wheat flours. Food Chem..

[32] Alonso-Miravalles L., O’Mahony J. (2018). Composition, Protein Profile and Rheological Properties of Pseudocereal-Based Protein-Rich Ingredients. Foods.

[33] Akharume F., Santra D., Adedeji A. (2020). Physicochemical and functional properties of proso millet storage protein fractions. Food Hydrocoll..

[34] Codină G.G., Zaharia D., Stroe S.G., Ropciuc S. (2018). Influence of calcium ions addition from gluconate and lactate salts on refined wheat flour dough rheological properties. CYTA J. Food.

[35] Sehn G.A.R., Nogueira A.C., Almeida E.L., Chang Y.K., Steel C.J. (2015). Fortification of wheat dough with calcium and magnesium ions affects empirical rheological properties. Cereal Chem..

[36] Inglett G.E., Chen D., Liu S.X. (2015). Physical properties of gluten-free sugar cookies made from amaranth-oat composites. LWT Food Sci. Technol..

[37] Aprodu I., Banu I. (2017). Milling, functional and thermo-mechanical properties of wheat, rye, triticale, barley and oat. J. Cereal Sci..

[38] Zaidul I.S.M., Yamauchi H., Kim S., Hashimotom N., Noda T. (2007). RVA study of mixtures of wheat flour and potato starches with different phosphorus contents. Food Chem..

[39] Ekpa O., Palacios-Rojas N., Rosales A., Renzetti S., Fogliano V., Linnemann A. (2020). Genotype selection influences the quality of gluten-free bread from maize. LWT Food Sci. Technol..

